# Genito-anal injury patterns and associated factors in rape survivors in an urban province of South Africa: a cross-sectional study

**DOI:** 10.1186/s12905-015-0187-0

**Published:** 2015-03-27

**Authors:** Ruxana Jina, Rachel Jewkes, Lisa Vetten, Nicola Christofides, Romi Sigsworth, Lizle Loots

**Affiliations:** School of Public Health, Faculty of Health Sciences, University of the Witwatersrand, Johannesburg, Gauteng South Africa; Gender and Health Research Unit, South African Medical Research Council, Pretoria, South Africa; Wits Institute for Social and Economic Research, Centre for the Study of Violence and Reconciliation, University of the Witwatersrand, Johannesburg, Gauteng South Africa; Institute for Security Studies, Pretoria, Gauteng South Africa

**Keywords:** Genital injury, Rape, South Africa

## Abstract

**Background:**

The prevalence of genito-anal injuries in rape survivors varies significantly and the factors associated with the absence of injuries are not well understood. This plays a major role in the conviction of cases as the absence of injury is equated with a lack of assault. In such cases, health care providers face major challenges in presenting and defending their findings. The aim of this paper is to describe the absence of genito-anal injuries by site in a group of rape survivors and to identify factors associated with the absence of these injuries.

**Methods:**

In a cross-sectional study rape cases reported to the police in one province in South Africa were randomly sampled using a two stage sampling procedure. Data were obtained on the survivor, the circumstances of the rape and the findings of the medicolegal examination. Descriptive statistics were conducted for the prevalence of genito-anal injuries by site and logistic regression models were built to identify factors associated with the absence of genito-anal injuries for all survivors and those reported to be virgins.

**Results:**

In the sample of 1472 women injuries ranged from 1% to 36%. No significant injuries were reported for 749 (51%) survivors. In the multivariable model there was a significantly lower odds of having no injuries in survivors who were virgins, those raped by multiple perpetrators and those examined by a doctor with additional qualifications. In the model for survivors who were virgins, those with disabilities had a greater odds of having no injuries while those between the ages of 8 and 17 years had a lower odds of having no injuries compared to survivors below four years of age.

**Conclusions:**

This study found that being a virgin, multiple perpetration rape and the examiner’s qualifications were significantly associated with the absence of genito-anal injuries. Health providers should thus be aware that in all other respects there was no difference in survivors who had injuries and those who did not. It is important to reiterate the message that the presence of injuries does not necessarily prove that rape occurred nor does the absence disprove the fact.

## Background

Transcripts of expert witness testimonies in court cases for rape show that the presence or absence of injuries and its interpretation is usually a subject of immense focus in cross-examination [1]. Health care providers, as experts, are required to use evidence related to injuries to interpret their findings when cross-examined in court [2,3] and must demonstrate that their testimony is based on theories that are preferably evidence-based and accepted by others in the field. In paediatric cases, a classification of injuries has been developed [4] but in adults, the interpretation of evidence is much more complex. This allows for health care providers to be challenged in court especially in cases where no injuries have been found as some literature have reported on the presence of injuries after consensual intercourse as well [5-9]. Higher rates of prosecution and conviction have been found in cases with injuries documented [10,11].

Globally, the reported prevalence of genito-anal injuries after rape is between 9% and 87% depending on the ages of the survivors in the sample, method of examination and the type of injuries included [6,8,9,12-28] while in South Africa genito-anal injuries have been reported in 7% to 58% of rape cases [29-33] and in 19% to 56% of rape homicides [34,35]. As not all women who report rape have injuries, understanding what factors are associated with injury patterns and what may influence these is potentially very valuable. Although there is some data available on the prevalence of genito-anal injuries after rape there has been much less analysis on the factors associated with these injuries. Studies have reported that age, race, educational level, postmenopausal state, previous sexual experience, previous experience with violence, parity, contraceptive use, alcohol usage prior to the rape, relationship with the perpetrator, location of the assault, reported anal penetration, threats of violence and type of resistance during the rape, the presence of non-genital injuries, time to examination, and levels of experience of examiners are significantly associated with the presence of genito-anal injuries in multivariable analysis, but these associations have not been consistent across studies [17-19,25,26,28,36-55].

None of the above-mentioned studies have been done in South Africa and as this country has a very high rate of rape homicide [35], there is reason to suspect that rape in South Africa may be more violent and different in some respects from rape elsewhere in the world. Given the dearth of literature in this area, there is a need to increase the pool of international evidence and more so that linked to the local context. The aim of this paper is to describe the prevalence of genito-anal injuries by site and identifies factors associated with the absence of genito-anal injuries in rape survivors in Gauteng, South Africa.

## Methods

A cross-sectional study was conducted that randomly sampled rape cases reported to the Gauteng province police in 2003. Police files including the medicolegal records (J88 form) [56,57] and court records were reviewed for cases selected that met the common law definition of rape during that period, which was to have “intentional, unlawful sexual intercourse with a woman without her consent” [58].

A two stage sampling procedure was used to select the cases to be reviewed. In the first stage, 70 police stations were sampled out of the 128 that had rape cases reported in 2003, using probability proportional to size. Here the total rape cases reported in the year were used. For the second stage of sampling, 30 cases (or all cases if there were less than 30 cases reported) were randomly selected from all cases that had been identified to be closed at the time of data collection in 2008. There were no replacements of cases if dockets pertaining to selected cases were not found. In total a sample size of 2,068 cases was obtained.

Data were obtained from the police dockets on the details of the survivor and her behaviour after the rape, circumstances of the rape, and details of the perpetrator. Closed questions were used for well-defined variables whereas open questions that were post-coded were used for information on the circumstances of the rape. The J88 forms were copied verbatim or photocopied and coded by three health care providers on the team. Here information was obtained on the examination that was conducted with the findings and conclusions of the health care provider. Fieldworkers were trained for a week prior to data collection and the consistency in data collection was maintained through supervision by two of the researchers and frequent discussions.

The data were analysed using Stata 10.0 with the svy command to adjust for the design of the study. For this study, the sample size was restricted to cases where an examination was conducted and a J88 form completed within 10 days of a rape, with vaginal penetration, being reported. Missing data for individual variables were excluded from the analysis. Eighteen women reported having anal penetration but they had all experienced vaginal penetration as well. Age was categorized into three groups: an under 18 group including children and adolescents, a group from 18 to 44 years, and a group of women 45 years and older to include postmenopausal women. Smaller age categories were used in the analysis for survivors who had no previous sexual intercourse experience to account for varying oestrogen levels at different ages [59].

The pattern of genito-anal injuries were initially described according to the type and location of the injuries as listed on the J88 form with some categories being merged (clitoris and frenulum of clitoris, urethral orifice and paraurethral folds, labia majora and labia minora, posterior fourchette, fossa navicularis, perineum, hymen, vagina and cervix, skin around the orifice, orifice and sphincter/anus). Health care providers are not required to report on their examination methods and techniques used on the J88 form and very few providers would do this on their own accord. According to a Department of Health manager of the service at the time, only one or two specialized facilities in the province were using toluidine blue dye or colposcopies for examination at the time. Vaginal and cervical findings would in the vast majority of facilities be identified with a speculum examination and where this section was not completed, it was assumed that the examination was not done. Anoscopic examinations are very rarely conducted for post-rape examinations and are not recommended with recent training, unless there is an indication for one to be done [60].

Considering the sensitivity and specificity of injuries in relation to rape, a combined variable was generated for genito-anal injuries. This included scratches, abrasions, tears, bruising, haematomas, and bleeding in the genito-anal area, hymenal clefts or swelling, cuts in the genital area and swelling in the anal area [61]. Redness, scars and pain were categorized as being uninjured. The absence of genito-anal injuries was then assessed for a number of subgroups, including those related to the survivor, factors pertaining to the rape, and health service factors. Chi Square and Fischer Exact tests were done to compare proportions by subgroup. A logistic regression model was built with the absence of genito-anal injuries as the outcome variable and the demographic characteristics of the survivors, circumstances of rape and health service factors as explanatory variables. Backward selection was used to build the final model while adjusting for age of survivors and time to examination as these were considered to have a potential confounding effect. A separate model was built for virgins using the same approach. In the analysis a p value of <0.05 was considered to be significant.

This study was approved by the Human Research Ethics Committee of the University of the Witwatersrand (M040331). Permission was also obtained from the South African Police Services and Department of Justice. All identifiers related to the survivor and service providers were excluded from the data collection.

## Results

There were 1472 women who had gynaecological examinations and findings reported on the J88 forms of which 371 had an anal examination as well. The majority of the survivors were between the ages of 18 and 44 years (n = 853, 59%) while 37% were less than 18 years of age (n = 532). About a third of the survivors were reported to be virgins at the time of the examination (n = 361, 27%) and there were very few survivors who were recorded to have disabilities (n = 25, 2%) or to have been under the influence of drugs or alcohol (n = 53, 5%). Most of the survivors were examined within 48 hours of the rape (n = 1056, 84%) and 19% (n = 269) reported being raped by multiple perpetrators.

Genital injuries ranged from 1% (n = 20) for cuts to 36% (n = 535) for scratches, abrasions or tears (Table 1). Most injuries were recorded on the hymen (n = 666, 45.2%) followed by the posterior fourchette (n = 451, 30.6%), labias combined (n = 235, 16.0%) and fossa navicularis (n = 217, 14.7%). Redness was the most common injury recorded for the clitoris and its frenulum, urethral orifice and paraurethral folds, and the labia majora and minora while scratches, abrasions or tears were the most common injuries recorded for the posterior fourchette, fossa navicularis and perineum. The skin around the orifice had the most number of injuries recorded on the anal examination (n = 38, 10.2%) with scratches, abrasions or tears being the most common injury recorded on anal examination (Table 2). Overall, 723 (49.1%) survivors were found to have genito-anal injuries.

**Table 1:** **Genital injuries by location**

**Gynaecological examination** ^**#**^	**Total (N = 1472)** ^*^	**Clitorus and frenulum (N = 80)** ^**#**^	**Urethral orifice and paraurethral folds (N = 139)** ^**#**^	**Labias majora and minora(N = 235)** ^**#**^	**Posterior fouchette (N = 451)** ^**#**^	**Fossa navicularis (N = 217)** ^**#**^	**Perineum (N = 79)** ^**#**^	**Hymen (N = 666)** ^**#**^	**Vagina (n = 188)** ^**#**^	**Cervix (N = 70)** ^**#**^
**Scratches/abrasions/tears**	535 (36.3%)	35 (43.8%)	65 (46.8%)	126 (53.6%)	364 (80.7%)	130 (59.9%)	62 (78.5%)	237 (35.6%)	97 (51.6%)	19 (27.1%)
**Cuts**	20 (1.4%)	1 (1.3%)	5 (3.6%)	8 (3.4%)	10 (2.2%)	5 (2.3%)	5 (6.3%)	1 (0.2%)	1 (0.5%)	0 (0.0%)
**Bruising/haematoma**	254 (17.3%)	32 (40.0%)	48 (34.5%)	75 (31.9%)	140 (31.0%)	100 (22.2%)	38 (48.1%)	119 (17.9%)	47 (25.0%)	15 (21.4%)
**Bleeding**	145 (9.9%)	14 (17.5%)	22 (15.8%)	29 (12.3%)	57 (12.6%)	15 (6.9%)	14 (17.7%)	58 (8.7%)	79 (42.0%)	52 (74.3%)
**Hymenal clefts**								63 (9.5%)		
**Hymen swollen**								138 (20.7%)		
**Scars from? assault**	78 (5.3%)	4 (5.0%)	11 (7.9%)	17 (7.2%)	66 (14.6%)	11 (5.1%)	5 (6.3%)	12 (1.8%)	1 (0.5%)	0 (0.0%)
**Redness/inflammation**	238 (16.2%)	62 (77.5%)	107 (77.0%)	139 (59.2%)	106 (23.5%)	86 (39.6%)	36 (45.6%)	24 (3.6%)	19 (10.1%)	10 (14.3%)
**Tenderness/pain**	76 (5.2%)	7 (8.9%)	6 (4.3%)	16 (6.8%)	16 (3.5%)	12 (5.5%)	6 (7.6%)	36 (5.4%)	51 (27.1%)	4 (5.7%)

^*^Total sample size includes all patients who underwent a gynecological examination.

^#^Total number of patients who had injuries recorded at a specific site, for which type of injuries are presented in the rows. Patients could have injuries at more than one site, and could have more than one type of injury per site. Each cell in the table represents the proportion of patients who had injuries reported of a specific type at a specific site.

**Table 2:** **Anal injuries by location**

**Anal examination** ^**#**^	**Total (N = 371)** ^*^	**Skin around orifice (N = 38)**	**Orifice (N = 29)**	**Sphincter/Anus (N = 16)**
**Scratches/abrasions/tears**	30 (8.1%)	23 (60.5%)	21 (72.4%)	9 (56.3%)
**Bruising**	10 (2.7%)	10 (26.3%)	4 (13.8%)	3 (18.8%)
**Bleeding**	2 (0.5%)	1 (2.6%)	2 (6.9%)	0 (0.0%)
**Swelling/tyre sign** ^**$**^	13 (3.5%)	13 (34.2%)	11 (35.5%)	3 (18.8%)
**Scars from ?assault/funelling** ^**^**^	7 (1.9%)	5 (13.2%)	6 (20.7%)	3 (18.8%)
**Redness/inflammation**	16 (4.3%)	16 (42.1%)	11 (37.9%)	7 (43.8%)
**Tenderness/pain**	3 (0.8%)	2 (5.3%)	2 (6.9%)	2 (12.5%)

^*^Total sample size includes all patients who underwent an anal examination.

^#^Total number of patients who had injuries recorded at a specific site, for which type of injuries are presented in the rows. Patients could have injuries at more than one site, and could have more than one type of injury per site. Each cell in the table represents the proportion of patients who had injuries reported of a specific type at a specific site.

^$^Perianal swelling in the form of a ring.

^^^Relaxation of the external anal sphincter but not of the internal anal sphincter which results in a funnel-like appearance of the anus on physical examination. Appears with repeated abuse.

On bivariate analysis (Table 3), age was found to be significantly associated with the absence of injuries. Among survivors without injuries, 30% were less than 18 years of age, whereas 44% of those with injuries were aged under 18 years. Other factors found to be significantly associated with the absence of injuries included survivors who had had a previous pregnancy (60% of those with no injuries had been pregnant versus 51% of those with injuries) and those who were raped by a perpetrator that was armed (39% of those with no injuries versus 34% of those injured). Furthermore, the percentage of virgins among those with injuries were significantly greater than that among those with no injuries (36% versus 20%). Similarly rape by multiple perpetrators (21% versus 16%) and being examined by a doctor with no additional graduate qualifications (16% versus 9%) was more common amongst those with injuries.

**Table 3:** **Absence of genito-anal injuries by factors related to the patient, rape and examination**

	**Total**	**Absence of genito-anal injuries (N = 749)**	**Presence of genito-anal injuries (N = 723)**	**p value**
**PATIENT INFORMATION**				
**Age (N = 1447)**				0.000
<18 years	532 (36.8%)	221 (30.0%)	311 (43.9%)	
18 - 44 years	853 (59.0%)	489 (66.3%)	364 (51.3%)	
≥45 years	62 (4.3%)	28 (3.8%)	34 (4.8%)	
**Virgin (N = 1311)**				0.000
No	950 (72.5%)	526 (80.4%)	424 (64.5%)	
Yes	361 (27.1%)	128 (19.6%)	233 (35.5%)	
**Previously pregnant (N = 1131)**				0.004
No	502 (44.4%)	243 (40.5%)	259 (48.8%)	
Yes	629 (55.6%)	357 (59.5%)	272 (51.2%)	
**Disability (N = 1472)**				0.354
No	1447 (98.3%)	734 (98.0%)	713 (98.6%)	
Yes	25 (1.7%)	15 (2.0%)	10 (1.4%)	
**Under influence of drugs or alcohol (N = 1114)**				0.835
No	1061 (95.2%)	536 (95.4%)	525 (95.1%)	
Yes	53 (4.8%)	26 (4.6%)	27 (4.9%)	
**RAPE**				
**Length of time perpetrator detained victim (N = 1411)**				0.224
Less than a day	1204 (85.3%)	620 (86.6%)	584 (84.0%)	
1 day of more	207 (14.7%)	96 (13.4%)	111 (16.0%)	
**Multiple perpetrators (N = 1437)**				0.038
No	1168 (81.3%)	616 (83.6%)	552 (78.9%)	
Yes	269 (18.7%)	121 (16.4%)	148 (21.1%)	
**Perpetrator victim relationship (N = 1414)**				0.320
Any relative	99 (7.0%)	46 (6.4%)	53 (7.7%)	
Curent or ex-partner	200 (14.1%)	113 (15.6%)	87 (12.6%)	
Stranger or someone known only slightly/by sight	697 (49.3%)	346 (47.9%)	351 (50.8%)	
Some other known person	418 (29.6%)	218 (30.2%)	200 (28.9%)	
**Perpetrator armed (N = 1442)**				0.039
No	917 (63.6%)	448 (61.1%)	469 (55.2%)	
Yes	525 (36.4%)	285 (38.9%)	240 (33.9%)	
**Victim hurt with weapon (N = 517)**				0.116
No	399 (77.2%)	226 (79.6%)	173 (74.3%)	
Yes	118 (22.8%)	58 (20.4%)	60 (25.8%)	
**Victim threatened (N = 1472)**				0.273
No	809 (55.0%)	401 (53.5%)	408 (56.4%)	
Yes	663 (45.0%)	348 (46.5%)	315 (43.6%)	
**Physical force used (N = 1429)**				0.192
No	557 (39.0%)	273 (37.4%)	284 (40.6%)	
Yes	872 (61.0%)	457 (62.6%)	415 (59.4%)	
**Victim resisted (N = 1472)**				0.857
No	809 (55.0%)	410 (54.7%)	399 (55.2%)	
Yes	663 (45.0%)	339 (45.3%)	324 (44.8%)	
**Number of penetrations (N = 1272)**				0.117
1	861 (67.7%)	465 (69.8%)	396 (65.4%)	
>1	411 (32.3%)	201 (30.2%)	210 (34.7%)	
**EXAMINATION**				
**Time from rape to examination (N = 1262)**				0.899
<2 days	1056 (83.7%)	528 (83.5%)	528 (83.8%)	
2 - 10 days	206 (16.3%)	104 (16.5%)	102 (16.2%)	
**Doctor's qualification (N = 1223)**				0.001
Medical degree	1072 (87.7%)	562 (91.5%)	510 (83.7%)	
Higher than medical degree	151 (12.4%)	52 (8.5%)	99 (16.3%)	

In the multivariable model of factors associated with the absence of injuries (Table 4) being a virgin (OR 0.4, 95% confidence interval (CI) 0.3 – 0.7), being raped by multiple perpetrators (OR 0.6, 95% CI 0.6 – 0.9) and being examined by a doctor with postgraduate qualifications (OR 0.6, 95% CI 0.4 – 0.9) were found to be significant. These factors were associated with a lower odds of having no injuries, in other words a greater likelihood of injury. Survivors who were examined 2 days or later after the rape were significantly more likely to have no genito-anal injuries recorded (OR 1.5, 95% CI 1.0 – 2.3). In the model for survivors who were virgins (Table 5), those with disabilities were found to have a greater odds of having no genito-anal injuries recorded (OR 4.9, 95% CI 1.2 – 20.1) while those between the ages of 8 and 17 years had a lower odds of having no genito-anal injuries (i.e. were more likely to be injured) compared to survivors less than 4 years of age.

**Table 4:** **Multivariable model to test the factors associated with the absence of genito-anal injuries**

	**Univariate analysis OR (95% CI)**	**p value**	**Multivariable analysis (N = 974) OR (95% CI)**	**p value**
**PATIENT INFORMATION**				
**Age (N = 1447)**				
<18 years	-			
18 - 44 years	1.9 (1.5 - 2.4)	0.000	1.4 (0.9 - 2.0)	0.157
≥45 years	1.1 (0.6 - 1.9)	0.835	0.5 (0.2 - 1.1)	0.078
**Virgin (N = 1311)**				
No	-	0.000	-	
Yes	0.4 (0.3 - 0.6)		0.4 (0.3 - 0.7)	0.000
**Previously pregnant (N = 1131)**				
No	-	0.011		
Yes	1.4 (1.1 - 1.8)			
**Disability (N = 1472)**				
No	-	0.329		
Yes	1.5 (0.7 - 3.6)			
**Under influence of drugs or alcohol (N = 1114)**				
No	-	0.945		
Yes	1.0 (0.5 - 1.8)			
**RAPE**				
**Length of time perpetrator detained victim (N = 1411)**				
Less than a day	-			
1 day of more	0.8 (0.6 - 1.1)	0.182		
**Multiple perpetrators (N = 1437)**				
No	-	0.022	-	
Yes	0.7 (0.5 - 1.0)		0.6 (0.4 - 0.9)	0.017
**Perpetrator victim relationship (N = 1414)**				
Any relative	-			
Current or ex-partner	1.6 (1.0 - 2.7)	0.062		
Stranger or someone known only slightly/by sight	1.2 (0.8 - 1.9)	0.356		
Some other known person	1.4 (0.9 - 2.2)	0.154		
**Perpetrator armed (N = 1442)**				
No	-			
Yes	1.3 (1.0 - 1.6)	0.042		
**Victim hurt with weapon (N = 517)**				
No	-			
Yes	0.7 (0.5 - 1.1)	0.124		
**Victim threatened (N = 1472)**				
No	-			
Yes	1.2 (0.9 - 1.4)	0.192		
**Physical force used (N = 1429)**				
No	-			
Yes	1.1 (0.9 - 1.4)	0.435		
**Victim resisted (N = 1472)**				
No	-			
Yes	1.0 (0.8 - 1.3)	0.898		
**Number of penetrations (N = 1272)**				
1	-			
>1	0.8 (0.6 - 1.0)	0.035		
**EXAMINATION**				
**Time from rape to examination (N = 1262)**			-	
<2 days	-		1.5 (1.0 - 2.3)	0.035
2 - 10 days	1.0 (0.7 - 1.4)	0.874		
**Doctor's qualification (N = 1223)**				
Medical degree	-		-	
Higher than medical degree	0.6 (0.4 - 0.9)	0.010	0.6 (0.4 - 0.9)	0.020

**Table 5:** **Multivariable model to test the factors associated with the absence of genito-anal injuries in survivors who were virgins**

	**Univariate analysis OR (95% CI)**	**p value**	**Multivariable analysis (N = 275) OR (95% CI)**	**p value**
**PATIENT INFORMATION**				
**Age (N = 349)**				
<4 years	-		-	
4 - 7 years	0.8 (0.3 - 1.8)	0.563	0.5 (0.2 - 1.5)	0.232
8 - 12 years	0.2 (0.1 - 0.6)	0.002	0.2 (0.1 - 0.5)	0.001
13 - 17 years	0.2 (0.1 - 0.6)	0.001	0.2 (0.1 - 0.4)	0.000
18 - 34 years	0.5 (0.2 - 1.6)	0.219	0.3 (0.1 - 1.1)	0.070
35 - 44 years	0.8 (0.0 - 17.4)	0.887	0.7 (0.0 - 15.3)	0.813
**Disability (N = 361)**				
No	-		-	
Yes	2.8 (0.9 - 8.8)	0.083	4.9 (1.2 - 20.1)	0.028
**RAPE**				
**Length of time perpetrator detained victim (N = 321)**				
Less than a day	-			
1 day of more	0.8 (0.4 - 1.6)	0.484		
**Multiple perpetrators (N = 342)**				
No	-			
Yes	0.8 (0.3 - 1.6)	0.424		
**Perpetrator victim relationship (N = 324)**				
Any relative	-			
Current or ex-partner	0.3 (0.0 - 1.5)	0.138		
Stranger or someone known only slightly/by sight	0.5 (0.2 - 1.0)	0.048		
Some other known person	0.9 (0.5 - 1.8)	0.769		
**Perpetrator armed (N = 339)**				
No	-			
Yes	0.5 (0.3 - 1.0)	0.064		
**Victim hurt with weapon (N = 59)**				
No	-			
Yes	0.5 (0.1 - 2.4)	0.355		
**Victim threatened (N = 361)**				
No	-			
Yes	0.7 (0.4 - 1.2)	0.202		
**Physical force used (N = 337)**				
No	-			
Yes	0.6 (0.4 - 1.0)	0.057		
**Victim resisted (N = 361)**				
No	-			
Yes	0.7 (0.5 - 1.2)	0.195		
**Number of penetrations (N = 274)**				
1	-			
>1	0.9 (0.5 - 1.8)	0.774		
**EXAMINATION**				
**Time from rape to examination (N = 281)**				
<2 days	-		-	
2 - 10 days	1.4 (0.8 - 2.4)	0.244	1.3 (0.7 - 2.4)	0.348
**Doctor's qualification (N = 310)**				
Medical degree	-			
Higher than medical degree	1.0 (0.5 - 2.0)	0.944		

## Discussion

This study adds important data on the prevalence and pattern of genito-anal injuries in the South African context. Of note, just over half of the survivors had no genito-anal injuries, and in these cases there is a possibility that this will be misconstrued by the police and the courts to mean that no rape occurred [62]. While this study did potentially include some cases of alleged rapes or false allegations, previous work in South Africa have shown that they make up a small proportion of between 1% and 3% of reported rape cases [29,63]. It is hoped that in rape cases where no injuries are reported, other supporting evidence will enable a conviction in court. Yet, it is noted that although convictions do occur when no injuries are recorded, it is quite low in South African courts, more so for adult survivors (9% conviction in cases with no reported injuries) than in children (33%) [11].

Work in the field has shown that genito-anal injury detection is complex, and providers are limited by the availability of the necessary equipment and resources to conduct examinations and identify injuries. This is a challenge in South Africa as very few facilities had colposcopies at the time of the study and anecdotal evidence from present day indicates that even when colposcopies are available, they are not always used by providers. Similarly, the usage of toluidine blue dye has been erratic with the vast majority of facilities not having the dye available. In addition, the J88 form does not specifically require providers to describe the examination techniques that they used, thus depending on astute providers filling in this information at their own discretion when necessary. In essence, this implies that there is probably significant under detection of injuries with only easily identifiable injuries being reported on and tested in this study, as opposed to all possible injuries.

In survivors with injuries, there was substantial variation in the prevalence of different injuries. Common injuries included scratches, abrasions, tears, bruising and haematomas. Redness was also frequently reported, yet it is a non-specific sign and can undergo much scrutiny in court especially if it is an isolated finding [61]. This is harder to defend on cross-examination as a finding indicative of sexual assault as there are a number of other common causes and we thus excluded it in our final analysis. The predominant locations of injuries were as expected [13], with most of the injuries reported on the hymen, posterior fourchette and fossa navicularis. Major issues of concern are the lack of uniformity in examining survivors, dependence on the skill and experience of the examiner and discrepancies in recording of injuries [7]. Defining the significance of injuries also remains a challenge because it is recognised that vigorous consensual sex can also result in injury [6,9]. There has been a call to develop a standardised classification system with an injury severity scale, yet it is clear that this will still not resolve the legal concern of whether rape occurred or not [7,13].

In previous studies, medical doctors and mental health practitioners have self-reported that training improved their clinical practice [64-66] and this was found in a small study with doctors which evaluated the completion of clinical notes and collection of evidence after training [67]. Studies conducted with nurses who underwent training for sexual assault examiner programmes have also reported on improved collection of forensic evidence [68]. Furthermore, a previous study in South Africa showed that training alone resulted in increased knowledge and confidence in doctors and nurses [69]. Additional training thus appears to play a significant role in improving the quality of services provided to survivors of rape, and it is therefore interesting that this study found that there was a significant association between doctors who had some advanced training and the recording on injuries on the J88 form.

A consistent finding with international literature was that there is a significantly greater odds of finding injuries in survivors who were virgins [25,37,38] unlike the number of perpetrators which was not found to be associated with reported injuries in previous studies conducted in other countries [25,38]. Multiple perpetrator rape is a common phenomenon in South Africa with a self-reported prevalence of 9% to 16% [70,71]. It has been well described in the broader context of sexual coercion and with varying motivations, however punishment has been found to be a driver of the rape in about one in three cases [71,72]. In such cases, an increase in violence is possible.

This study found that children between the ages of 4 and 12 years were more likely to be injured compared to children of less than 4 years of age. This goes against a widespread perception that injuries would be more commonly found in very young children but fits with argument that is made that high levels of oestrogen which are present in girls under 5 can protect against injury in rape, whereas the changes in physiology and oestrogen levels occurring after 5 years of age results in heightened vulnerability [73].

By the same token, although it is reported that people with disabilities are at high risk of being sexually assaulted or abused [74], there were very few survivors with disabilities in this study. It may however reflect the difficulties these survivors face in verbalising and reporting abuse [75]. In survivors who were virgins, those with disabilities were found to have a significantly greater odds of having no injuries recorded. No other studies were found that described in detail injury patterns in rape survivors with disabilities.

Limitations of the study included having absent J88 forms, which occurred in 23% of the main sample of dockets. In addition, only rape cases that were reported to the police were included in the study, and this may reduce the generalisability of and bias findings, as survivors in certain relationships (e.g. rape in marriage) or those lacking injuries may be less likely to report their cases. On the other hand, cases of false allegations and alleged rape may also be included in the study, however it is assumed that the proportion would be small. The study was limited to survivors who were examined within 10 days of the rape as it was assumed that there would be very few significant genito-anal injuries visible beyond this period but this could have differentially excluded some groups of survivors e.g. young children who would be more likely to present outside this time period. Recorded injuries on the J88 form may not be indicative of actual injuries, for example it is unclear why there are a substantial proportion of bruises reported on the vagina, but this does reflect actual practice in the province and the findings that are presented in court. The examination techniques used is not recorded on the J88 forms but only a couple of specialized facilities in Gauteng province had colposcopies or toluidine blue dye available in 2003. Gauteng, however, is fairly urban and thus findings may differ in more rural areas of the country. In identifying factors associated with the absence injuries, explanatory variables were limited to what was recorded and available in the docket and on the J88 form.

## Conclusions

This study provides valuable data that health care providers can use during presentation in court as expert witnesses. Almost half of the survivors did not have any injuries recorded on the examination and hypothetically there are a number of factors that could influence the absence of injuries. This study found that being a virgin, multiple perpetration rape and being examined by a doctor with only basic qualifications were significantly associated with the absence of genito-anal injuries. Health providers as expert witnesses in court should thus be aware that in all other respects there was no significant difference in survivors who had injuries and those who did not. It is thus important to continuously reiterate the message that the presence of injuries does not necessarily prove that rape has occurred, especially taking into consideration the sensitivity and specificity of particular injuries, and similarly the absence of injuries do not disprove the fact [49]. The need to increase the utilization of specialized examination techniques through the availability of resources and training is necessary and could aid in the prosecution of rape in South Africa.

## Abbreviations

CI: Confidence interval
OR: Odds ratio
